# Spatiotemporal effects on dung beetle activities in island forests-home garden matrix in a tropical village landscape

**DOI:** 10.1038/s41598-021-96831-5

**Published:** 2021-08-30

**Authors:** G. Asha, K. Manoj, P. P. Megha, Palatty Allesh Sinu

**Affiliations:** Central University of Kerala, Periya, 671316 Kerala India

**Keywords:** Biodiversity, Tropical ecology, Conservation biology

## Abstract

Insects in seasonal tropics experience a wide range of temperatures along seasons, habitats, and a day. Therefore, the thermal tolerance of the insects can be a major driver for their habitat preference, temporal patterns of activity, and formation of communities. We examined the dung beetle communities of eleven pairs of neighboring open (home gardens) and closed habitats (sacred groves) during dry and wet seasons and diel periods (day and night) to understand the dung beetle activities along a spatiotemporal gradient constituted by the sacred groves—home garden matrix on a tropical village landscape. We tested the following hypotheses: (i) closed habitats have greater activities of dung beetles over open habitats; (ii) the diurnal communities of dung beetles are different from the nocturnal communities; and (iii) the diurnal-nocturnal activities of dung beetles could be predicted by the habitat and season. We considered abundance, richness, total biomass, and Shannon diversity of overall beetles, abundance of different functional groups, and species composition in communities as the quantitative measures in the predictive statistical models. In total, 2727 dung beetles belonging to 38 species, ten genera, and three functional groups were collected. The open habitat supported more number of dung beetles (N = 2318) than the closed habitat (N = 409). The diurnal communities were different from nocturnal communities, particularly in open habitat, where the temperature was different between day and night. The dominant species of the diurnal communities of open habitat hardly used the closed habitat in any context including dry–wet seasons, but the nocturnal communities of the open habitat were closer to the communities of closed habitat. The diel period and habitat predicted the abundance activity of functional groups; season was a poor predictor of dung beetle activities. Given that the species composition has turned over across habitats, and the closed habitat supported remarkably lesser number of beetles than the open habitats, the closed habitat is unlikely to be a thermal refuge for the open habitat species in village landscapes that have island forests, such as sacred groves, and home gardens form a matrix.

## Introduction

The global climate changes in the Anthropocene period dramatically modify habitat, microhabitat, and niche of species and shape the spatiotemporal activities of species and communities^1^. Among various climatic variables, temperature and solar incidence are crucial for physiological activities, life histories, and growth rates of insect species^2–4^. They are critical for predicting abundance, richness, and composition of species in various ecological communities across geographical locations, habitats, microhabitats, seasons, and diel periods^5^. As a result, the habitats may interact with seasons and diel periods to affect the species performance and abundance. Studying the effect of season and diel period on species abundance is central to understand how insects share a habitat or microhabitat for using resources effectively and how they form ecological communities. While the effect of season as a temporal factor on species distribution has been researched well, the effect of the diel period, as a temporal factor, on distribution, ecological performance, persistence, abundance, and composition of species in ecological communities is unknown or known only for certain geographical locations.

Dung beetles—a prevalent indicator taxon—respond differently with the habitat type and season across geographical regions, latitudes, and altitudes^6–10^. Studies have shown that the pastures that have been used for cattle grazing in tropics and subtropics are the poor habitats for the dung beetles, habitats that maintain some canopy, such as plantations and orchards, have moderate diversity of the beetles, and the closed forests have the highest diversity of the beetles^11–15^. In contrast, some other studies have shown that the open habitat, such as the grasslands used by the browsing wild ungulates in the tropics, have the highest abundance and taxonomic diversity of dung beetles, and the closed forests are the poor habitat for dung beetles^16–18^.

While the dung—the ephemeral resource—seems the most important predictor of species distribution and abundance of dung beetles, the habitat variables including temperature and canopy closure level are likely to predict the diel activity of dung beetle communities in habitats and seasons^19^. Recently, studies have demonstrated that the dung beetle species have different degrees of tolerance to temperatures^20–23^. Their flight and physiological activities can be affected by the ground-level temperature^24^. Diurnal species inhabiting open habitats exhibit lower endothermy than those inhabiting habitats with closed canopy cover, whereas nocturnal species exhibit similar endothermy in both the types of habitats^25^. Therefore, the diel activity is prominent among dung beetle species^24,26,27^. In the current scenario of global climate changes, it is vital to have information of how species might respond to temperatures and use habitats and microhabitats for foraging resources and other physiological activities to understand how species are coping with this global phenomenon.

In the tropics, where the dung beetles are species-rich and diverse, temporal distribution appears more relevant for their coexistence^28,29^. However, studies on the temporal distribution of dung beetle species in tropical forests are scarce or limited to only certain geographical regions (see Feer and Pincebourde^29^, Niino et al.^30^). Recent studies have unraveled certain habitat- or geographical region-specific patterns for dung beetle species distribution in habitats and microhabitats across different diel periods. One proposal was that the nocturnal community of species of closed habitats could be a subset of the diurnal community of open habitat species in a tropical high altitude forest fragment-grassland system^31^. The other finding was that the diurnal beetles, due to thermoregulatory constraints, could be smaller and metallic, and the nocturnal species could be larger and black^30,32,33^. Gimenez-Gomez et al.^25^ suggest that the diurnal species in the open habitats have smaller size and low endothermy than the related nocturnal species. The dung beetles have three functional groups based on managing dung—rollers, tunnellers, and dwellers, which might respond differently to the diel periods and habitat types^19,34^.

In the present study, we examined whether (i) the diurnal community of dung beetles was different from the nocturnal community, and (ii) the diel activity of dung beetles was affected by the habitat and the season. We hypothesized that if the season, habitat, and diel periods are the major determinants of the activity and distribution of dung beetles, their effect should be observed along the gradients of these three factors, and the diurnal composition of dry periods and open habitats should be distinct from the communities of wet periods and closed habitats.

## Methods

### Study area

**Figure 1:**
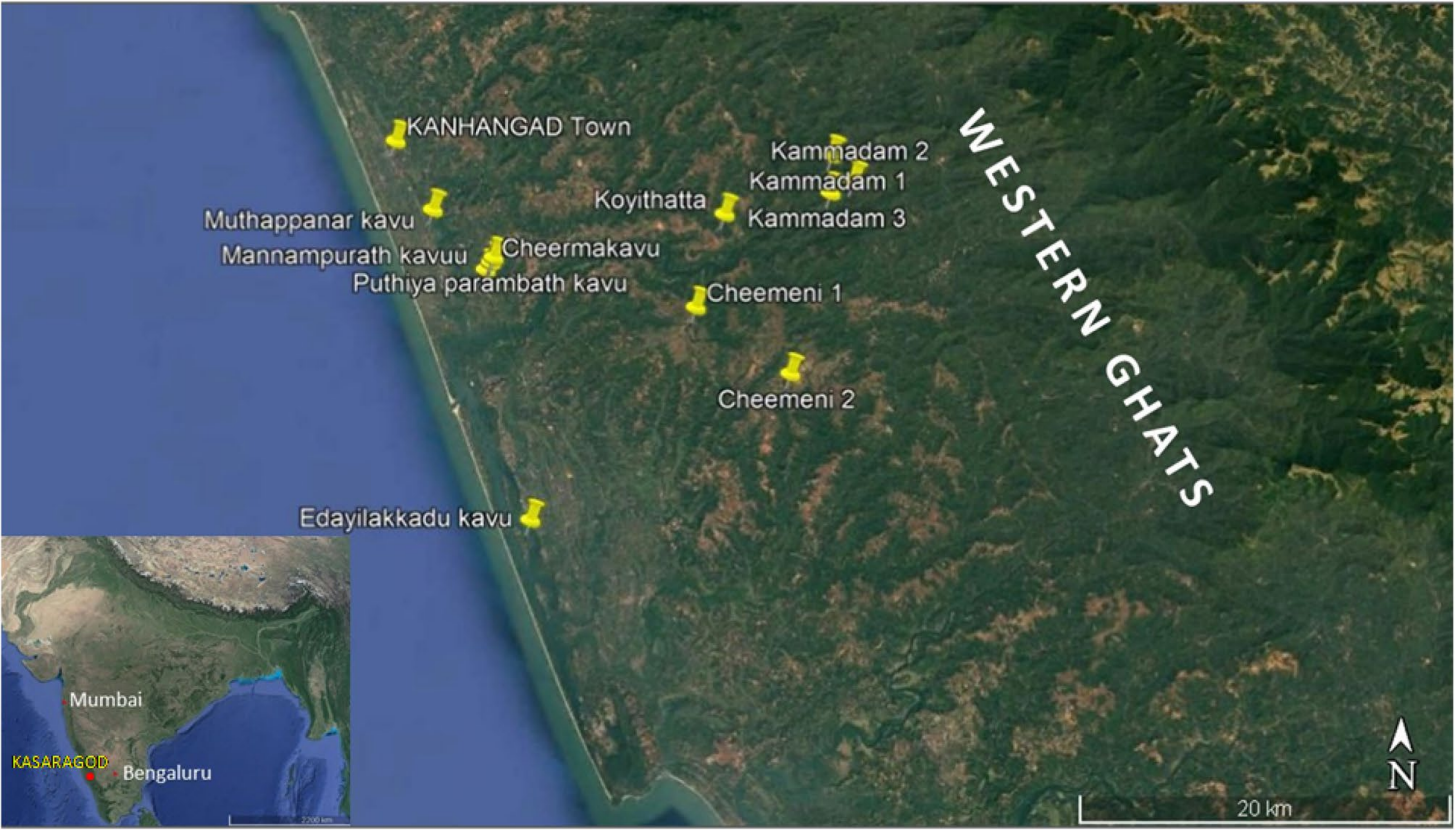
Google earth image of the study sites and Indian peninsula (inset). Copyright: GOOGLE EARTH PRO.

The study was conducted in an anthropogenic village landscape constituted by the mosaic of agro-landscape, human settlements, and relics of natural forests that form into island forests—the sacred groves (SG)—on the lowlands close to the western boundaries of the Western Ghats biodiversity hotspot in the north Malabar region (Kasaragod district) of Kerala state in peninsular India (12.5080°N, 74.9882°E) (Fig. 1). Sacred groves—the spiritual hubs of the Hindu community at present—were the self-enforced spiritual institutions during the period of animism^35,36^. They are protected either by joint Hindu families, temple trust, or local government bodies such as panchayats or municipalities. The neighborhood community worships both the local deities and *Sanskritized* gods in these groves. At present, SGs of the study region contain small patches of semi-evergreen forests that forbid entry of people for recreation or resources for livelihood support. For that reason, they are well protected and experience fewer disturbances from local neighborhood communities. They receive local vigilance from local communities. They have a thick bed of continuous leaf litter on the forest floor and a continuous closed canopy. They are also important centers of insect diversity, including insect functional diversity^35,37,38^. The sources of natural dung in SGs include macaques (*Macaca radiata* E. Geoffroy Saint-Hilaire, 1812), Brown-Palm Civets (*Paradoxurus hermaphroditus* Pallas, 1777), bats (*Pteropus* spp Brisson, 1762), wild boars (*Sus scrofa* Linnaeus, 1758), and sometimes the free-ranging livestock animals, such as cattle, buffalo, goats, dogs, cats, and even chicken (personal observation).

**Figure 2:**
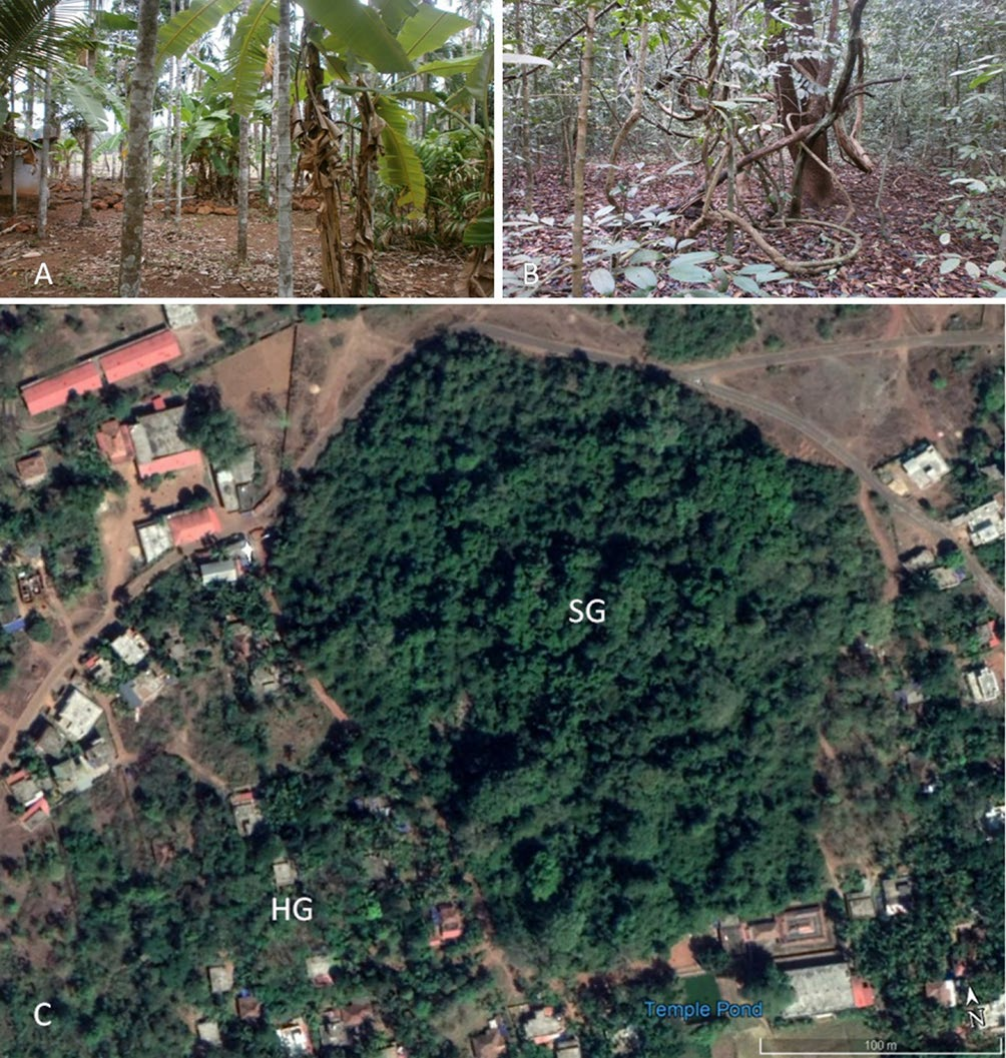
(**A**) Closer view of a home garden, (**B**) a sacred grove, and (**C**) satellite view of a study site (Cheemeni 1). Photo credits for (**A**,**B**): G. Asha; for (**C**). Copyright: GOOGLE EARTH PRO.

The home gardens (HGs) that surround the SGs are constituted primarily by the coconut palms at the upper story in the study area (Fig. 2). They are a relatively open habitat (canopy cover = 27.7 ± 8.8%) to the SGs (canopy cover = 71.8 ± 6.8%). The average light intensity (Lux) in SG and HG was 600 and 2300, respectively. The floor of the HGs was bare and exposed to the direct sunlight, but the floor of SGs was covered by leaf litter (litter depth = 4.5 ± 1.2 cm). The soil types of both the habitats were clay, clay loam, or sandy clay loam type.

The sites experience typical tropical coastal weather with a clearly defined dry period and wet period. The dry period (March–May) records a maximum temperature of 34 °C during the day and 30 °C during the night. The wet period (June–September) records a maximum temperature of 29 °C during the day and 25 °C during night. The winter (December–February) is mild with an average maximum day temperature of about 29 °C and an average minimum night temperature of about 23 °C (Courtesy: Regional Agricultural Station, Pilicode). The sites also receive 2656 mm rainfall during the southwest monsoon and about 40 mm rainfall during the northeast monsoon (October–December).

### Sampling

We selected the two habitats in a paired manner. We selected an HG on the longest edge of the adjoining SG for sampling. A site, therefore, contains an SG (closed fragment of the forest)–HG (relatively open habitat) pair. We selected 11 such sites located at ca. 10–133 m a.s.l. and at a mean aerial distance of about 21 km among them. The selected SGs of the present study had a median forest cover area of 0.018 km^2^ (range = 0.01–0.28 km^2^) (Table S1). This design of paired habitats in sites yields an independent sample of dung beetles and allows for studying the specific effect of habitat on the diel activity of dung beetles by controlling the apparent effect of space.

We used 1000 g fresh cow dung as baits to sample dung beetles from each habitat. The authorities of some SGs strictly instructed us to use only cow dung for sampling beetles from SGs due to its sacred nature. We installed one such dung pad per habitat. We kept the dung on an undisturbed floor for allowing the soil to affect the dung beetle species and functional guilds in dung. To reduce the probability of losing roller beetles, we used five guarding pitfall traps around dung pads at a radial distance of 30 cm from the edge of the dung pad, which considerably reduced the probability of missing roller dung beetles when using dung pad alone for sampling dung beetles^39^. The dung pads on the ground simulate a natural condition and provide the arriving dung beetles to form realistic ecological communities by allowing some of them to dwell on dung and others to tunnel or roll the dung balls, controlled by the facilitation and competition among species for the resources. The dwellers, in particular, might even control the abundance of the subsequent dwellers and other functional guilds in real dung pads through competition^19,39,40^. Our study also involves analyzing the communities of overall dung beetles and the abundance of specific functional guilds of both habitats. The present methodology differs from that of most of the studies cited herein, which used dung-baited pitfall traps, and the abundance data of dung beetles in particular, therefore, might be different from those studies. The pitfall traps were plastic containers (ten cm diameter and 20 cm depth) flushed in soil with the mouth opening at ground level. We used 70% ethanol in the pitfall traps to preserve the beetles. One dung pad with five guarding pitfall traps makes it into a sampling unit. The sampling units in HG and SG were installed 100 m inside the respective habitats from the edge between SG and HG pair. The diurnal and nocturnal sampling in the two habitats in a paired site was carried out simultaneously. To study the effect of season on the dung beetle activities in habitats and along diel periods (day and night), sampling was performed during the dry period (March–April) and wet period (July) of 2018.

In each site, we operated the sampling units twice a day, each lasts for 12 h—(0600–1800 h and 1800–0600 h the next day)—for studying diurnal and nocturnal activities of the beetles. However, the second sampling units were installed ten m away from the first in each habitat as the pitfall traps and soil under the dung pad were flushed out for sorting beetles of the first sampling unit. The soil of one foot deep or to the level of tunnels of tunneller beetles under the dung pads was collected to recover the beetles from tunnels. To sort the beetles out from dung and soil, they were soaked in a bucket full of water and stirred well to allow the trapped beetles in dung and soil to float over the water. The beetles from pitfall traps were also sorted out. They were preserved in 90% ethanol, identified to species using Arrow (1931)^41^, and deposited in the entomological collection of the Department of Animal Science at the Central University of Kerala.

We measured the soil surface temperature using a HOBO environmental data logger. The temperature was measured on an hourly interval in both habitats. The soil surface temperature was different between habitats (*F*_1,80_ = 27.86, *p* < 0.00005), diel periods (*F*_1,80_ = 15.43, *p* = 0.0001), seasons (*F*_1,80_ = 79.01, *p* < 0.00005), and in habitats by diel periods (*F*_1,80_ = 6.19, *p* = 0.01) and in habitats by seasons (*F*_1,80_ = 4.13, *p* = 0.045). The HGs were warmer than SGs during dry and wet periods. The day temperature in HG was different from SG both during dry (31.3 °C vs. 28.6 °C; *p* = 0.007) and wet periods (29.03 °C vs. 26.97 °C; *p* = 0.005). However, the night temperature of the two habitats was alike in dry (30.1 °C vs. 28.74 °C; *p* = 0.06) and wet periods (26.2 °C vs. 25.9 °C; *p* = 0.4).

### Functional attributes of dung beetles

All species were grouped according to generic (*Onthophagus* or non-*Onthophagus*) and tribal memberships (Onthophagini, Oniticellini, or Miscellaneous tribes), dung management type (roller, tunneller, or dweller), and diel activity (diurnal or nocturnal beetles). Following our personal experience with the species and Arrow (1931)^41^ and Sabu et al. (2006)^42^, we attributed species to different nesting habits. We measured the length of species using an mm ruler and grouped the beetles into either small (< 1 cm) or big (> 1 cm). We calculated the mean biomass of beetles in each sampling unit. For that, we dried the beetles in an oven for 48 h at 40 °C and weighed them using an electronic weighing balance (Shimadzu Type no: BL220H). Seven species had less than five beetles in the sample. To take measurements of those species, we used beetles from the museum collections of the Department of Animal Science at the Central University of Kerala. We found the mean dry biomass of each species, which we multiplied with its abundance in a sample to find out species biomass. We added the species biomass in a community to find the total biomass of dung beetle communities in a particular dung pad following Beiroz et al. (2017)^43^.

### Data analysis

We identified indicator species for habitat using IndVal^44^. It gives a measure of species specificity and fidelity to an ecological state. Species with high specificity and high fidelity have a high indicator value^45^. It was calculated using the function ‘multipatt’ with 999 permutations in the R-package ‘indicspecies’. Dung beetle abundance matrix was used to find the indicator species; species with significant (*p* < 0.05) IndVal results were regarded as indicator species.

To estimate for the sampling completion, we used a sample-based coverage estimator of species richness for each habitat using iNEXT^46^. We had species richness, abundance, Shannon–Wiener diversity index, and total biomass of species and abundances of the above-mentioned functional groups to respond to diel activity, habitat, and season. We used Generalized Linear Mixed Effect Models (GLMMs) to estimate whether the response variables were significantly predicted by habitat, season, diel activity period, and the interaction among those factors. We used negative-binomial distribution as the error type in the models when the abundance and richness of the beetles were the response variables. We used Gaussian distribution as the error type in Linear Mixed Effect Models when Shannon–Weiner diversity and total biomass were the response variables. Although habitats, seasons, and diel activity periods can have similar richness, abundance, and species diversity, the communities can be constituted by different species. To examine for the changes in community composition of dung beetles between habitats, seasons, and diel periods, we calculated the group position by Permutational Multivariate Analysis of Variance (PERMANOVA) and the group dispersion by PERMDISP on a similarity matrix of species based on the Jaccard coefficient using the ‘adonis’ function of R-package ‘vegan’^47^. The results were graphically illustrated in multi-dimensional scaling (PCoA) using ‘capscale’ function available in the package ‘vegan’. We also generated heatmaps using the R-package ‘gplots’ to identify species communities and illustrate habitat similarity on species composition. Mean ± SD has been reported throughout the text unless mentioned otherwise.

## Results

### Species diversity

We sampled a total of 2727 individuals representing 38 species, ten genera, and three nesting guilds from both the habitats. The sample coverage was 0.9970 and 0.9952, respectively, for SG and HG. Thirty-six species were sampled from HG, and 24 species were sampled from SG. Out of these, fourteen and two species were sampled only from HG and SG, respectively. A total of 409 individuals were collected on SG, and 2318 individuals were collected on HG.

In total, both day (30 species) and night (28 species) sampled a more or less similar number of species, but night sampled more number of singleton species (*N* = 14) than the day (*N* = 9). We sampled 1645 beetles during the day and 1082 individuals during the night. The abundance and richness of nocturnal and diurnal dung beetles were different between habitats (Table 1). Wet season sampled 32 species and 1563 individuals, and dry season sampled 25 species and 1164 individuals. In habitats, one to nine species (5.81 ± 2.18) and four to ten species (7.72 ± 2.24) were collected in SG and HG, respectively, during the dry season. In the wet season, one to eighteen species (4.09 ± 2.25) and four to nineteen species (10.09 ± 4.72) were collected in SG and HG, respectively. We also sampled between 9 to 53 individuals (25.45 ± 13.67; SG) and 6 to 235 individuals (80.36 ± 77; HG) during the dry season and between 1 to 25 individuals (11.27 ± 8.47; SG) and 42 to 418 individuals (130.36 ± 116.35; HG) during the wet season in habitats (Table S2). Among the three nesting habits, the tunneller was species-rich and abundant in dry (20 species; 654 individuals) and wet seasons (28 species and 1144 individuals).

**Table 1:** Dung beetle abundance and richness across habitats (HG and SG), diurnal-nocturnal periods (D and N), and seasons (Dry and Wet). *HG* home garden, *SG* sacred grove, *D* day, *N* night.

Season	Habitat	Diel period	Total species (mean ± SD)	Total abundance (mean ± SD)	Tunneller abundance	Roller abundance	Dweller abundance
Dry	HG	D and N	22 (7.72 ± 2.24)	884 (80.36 ± 77)	429 (39 ± 32.51)	2 (1 ± 0)	453 (56.62 ± 76.42)
		D	15 (5 ± 3.23)	620 (62 ± 85)	166 (16.6 ± 16.07)	2 (1 ± 0)	452 (56.5 ± 76.48)
		N	13 (3.90 ± 2.38)	264 (24 ± 34.57)	263 (23.90 ± 34.59)	0	1
	SG	D and N	20 (5.81 ± 2.18)	280 (25.45 ± 13.67)	225 (20.45 ± 13.17)	40 (13.33 ± 12.66)	15 (2.5 ± 1.76)
		D	16 (2.8 ± 1.61)	89 (8.9 ± 11.27)	35 (3.5 ± 5.16)	40 (13.33 ± 12.66)	14 (2.33 ± 1.5)
		N	12 (3.63 ± 2.01)	191 (17.36 ± 14.64)	190 (17.27 ± 14.75)	0	1
Wet	HG	D and N	32 (10.09 ± 4.72)	1434 (130.36 ± 116.35)	1017 (92.45 ± 66.88)	0	417 (59.57 ± 112.60)
		D	24 (6.63 ± 3.38)	899 (81.72 ± 116.02)	485 (44.09 ± 41.80)	0	414 (69 ± 119.26)
		N	21 (5.7 ± 3.26)	535 (53.5 ± 53.17)	532 (53.2 ± 53.33)	0	3 (1.5 ± 0.7)
	SG	D and N	18 (4.09 ± 2.25)	129 (11.27 ± 8.47)	127 (11.54 ± 8.25)	0	2 (1 ± 0)
		D	10 (2.2 ± 1.92)	37 (4.11 ± 4.01)	36 (4 ± 3.74)	0	1
		N	15 (3.09 ± 1.3)	92 (8.36 ± 7.95)	91 (8.27 ± 7.78)	0	1

The IndVal results suggest that six dung beetle species were the indicator species of HGs—*Caccobius vulcanus* Fabricius, 1801 (*p* = 0.001), *Onthophagus fasciatus* Boucomont, 1914 (*p* = 0.003), *Tibiodrepanus setosus* Wiedemann, 1823 (*p* = 0.001), *Onthophagus centricornis* Fabricius, 1798 (*p* = 0.003), *Caccobius meridionalis* Boucomont, 1914 (*p* = 0.002), and *Tiniocellus spinipes* Roth, 1851 (*p* = 0.032). All these indicator species were smaller beetles (4.72 ± 1.22 mm), and collected abundantly during day and rarely at night. However, no species could be identified as an indicator species of SGs (Table S3).

### Effect of habitat, season, and diel activity period on dung beetle activities

**Figure 3:**
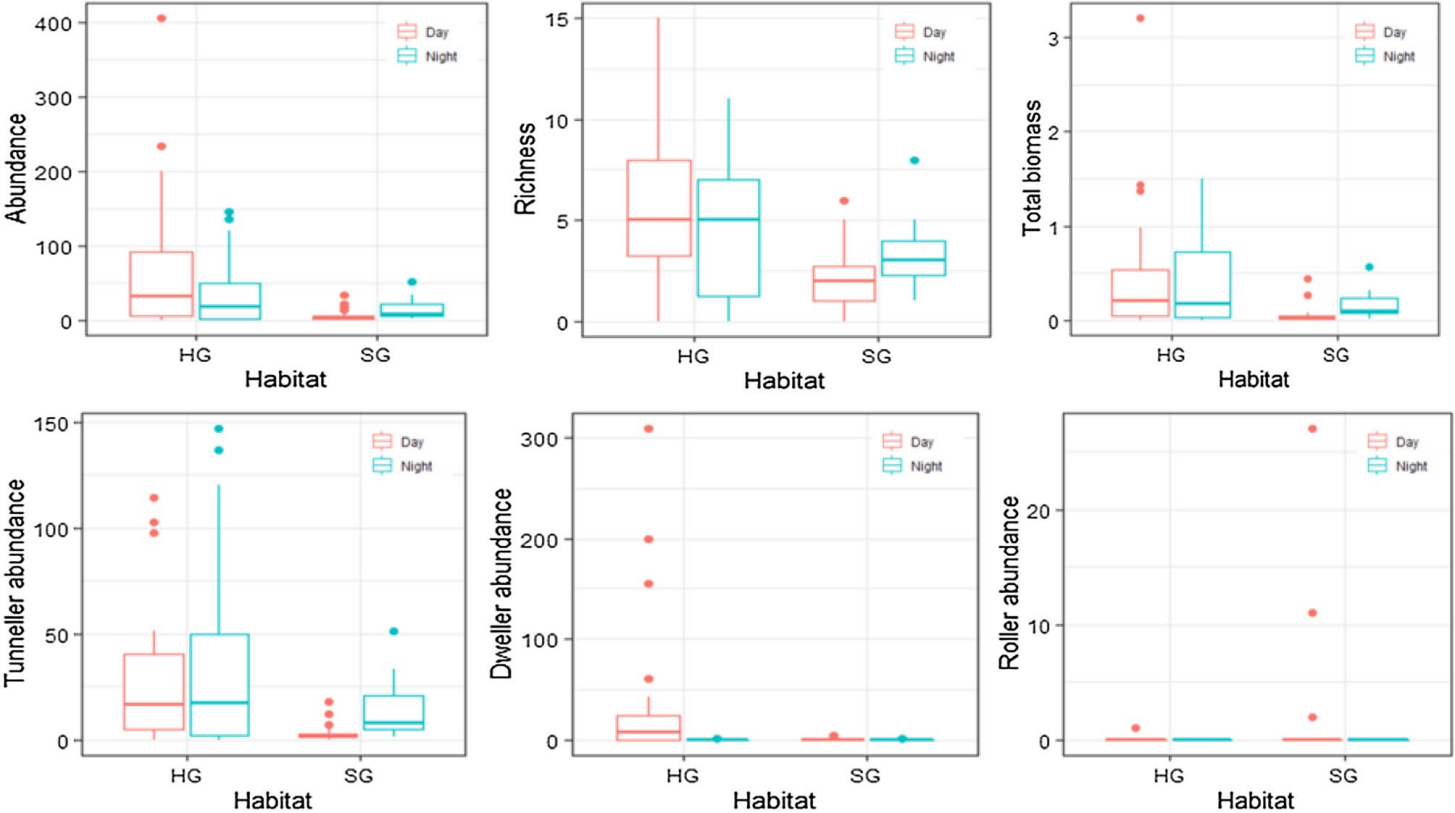
Diel activity of dung beetles and their functional groups in sites of closed (SG) and open (HG) habitats.

**Figure 4:**
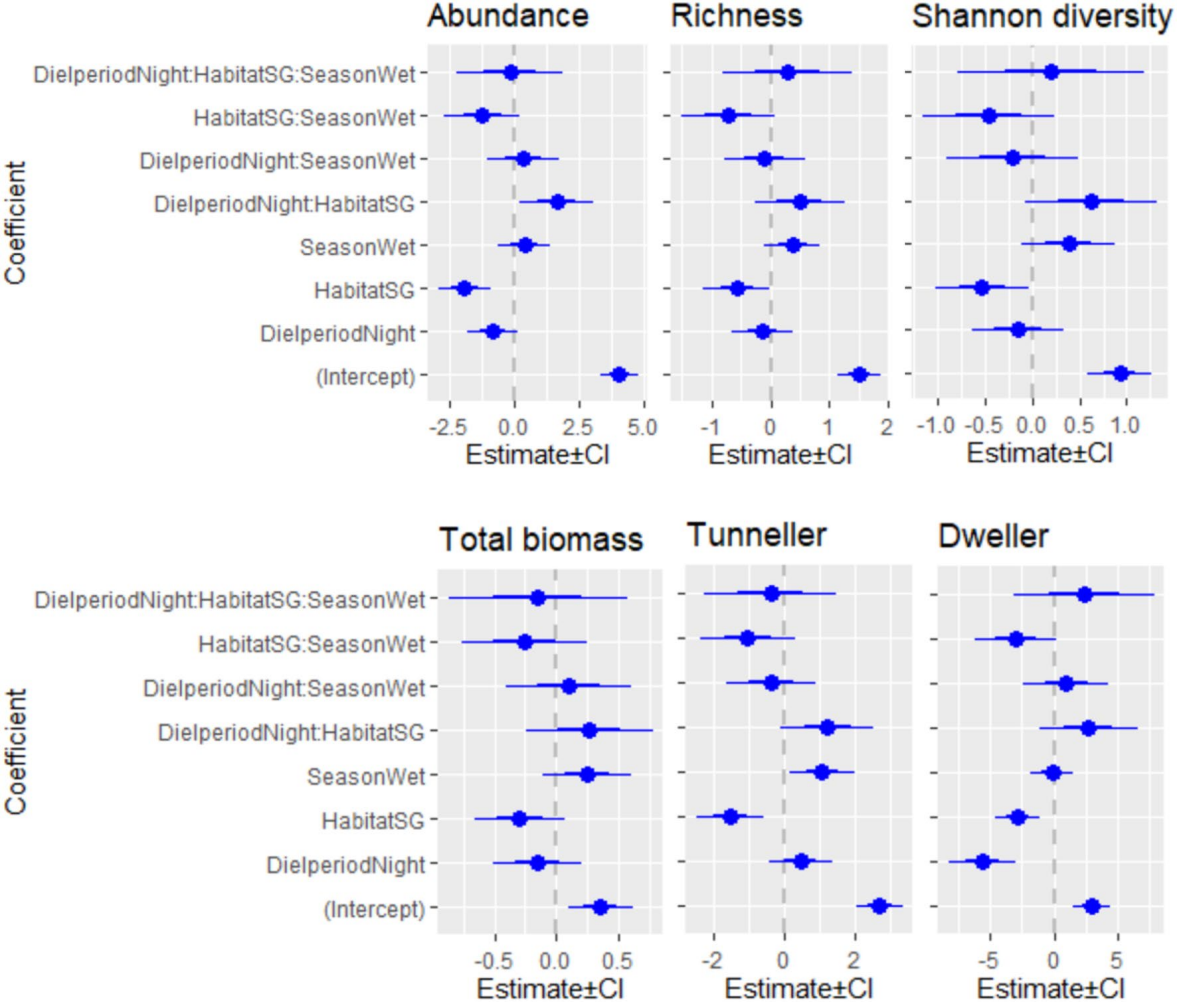
The coefficient plots show the effects of spatiotemporal factors on dung beetle activities, such as abundance, richness, Shannon diversity, total biomass of dung beetles, and abundances of tunneller and dweller beetles. The predictive models have diel period (Day and Night), habitat (HG and SG), season (Dry and Wet), and their interactions as the predictors. In the plots, the estimated value is showed by circle, standard error of the estimate by thick bars, and 95% CI by thin lines. The coefficients that have their SE of the estimate not touching the dashed lines are significant.

The abundance (− 1.94 ± 0.51, z = − 3.83, *p* = 0.0001), richness (− 0.58 ± 0.28, z = − 2.08, *p* = 0.04) and Shannon diversity (− 0.54 ± 0.24, z = − 2.18, *p* = 0.03) of overall dung beetles, and the abundance of tunnellers (− 1.56 ± 0.48, z = − 3.2, *p* = 0.001), dwellers (− 2.86 ± 0.89, z = − 3.22, *p* = 0.001) and rollers (2.96 ± 0.007, z = 400.1, *p* < 0.00005) were different between habitats (Fig. 4). The diel period, despite was a poor predictor of abundance (− 0.85 ± 0.49, z = − 1.71, *p* = 0.08), richness (− 0.14 ± 0.25, z = − 0.56, *p* = 0.57), Shannon diversity (− 0.16 ± 0.24, z = − 0.6, *p* = 0.5), and total biomass of dung beetle community (− 0.15 ± 0.18, z = − 0.86, *p* = 0.4), was an important determinant of the activities of dweller- (− 5.6 ± 1.3, z = − 4.3, *p* < 0.0005) and roller beetles (− 20.12 ± 0.007, z = − 2717.6, *p* < 0.00005) (Figs. 3, 4). The diel period also interacted with the habitat to affect the overall abundance of dung beetles (1.62 ± 0.71, z = 2.26, *p* = 0.02). In HG, the diurnal and nocturnal abundance of dung beetles was not different (− 0.64 ± 0.46, z = − 1.39, *p* = 0.16). However, the nocturnal abundance of dung beetles was higher than the diurnal abundance of dung beetles in SG (0.81 ± 0.35, z = 2.32, *p* = 0.02). The dwellers were predominantly collected in HG, but collected relatively poorly during the night both in HG (− 5.38 ± 0.50, z = 10.73, *p* < 0.00005) and SG (− 2.01 ± 0.76, z = − 2.66, *p* = 0.007). Rollers were represented only by two species and 42 individuals in the sample. Except for the two individuals (one each of *Sisyphus neglectus* Gory, 1833 and *Sisyphus longipes* Olivier, 1789) that were collected in HG, all remaining 40 individuals were collected in SG. *Sisyphus longipes*—a dominant roller species in the sample—was active during day hours and dry season. In HG, dwellers dominated the dung during the day, and the tunnellers dominated during the night. In SG, tunnellers dominated the dung both during day and night, but more in numbers during the night (1.37 ± 0.33, z = 4.11, *p* < 0.0005).

The season was a poor predictor of dung beetle activities (Fig. 4); however, the abundances of rollers (− 26.94 ± 0.007, z = − 3639.6, *p* < 0.00005) and tunnellers (1.07 ± 0.45, z = 2.36, *p* = 0.018) were affected by the season (Fig. 4). While the rollers were sampled only during the dry season, the tunnellers were sampled mainly in the wet season. The interaction of habitat and season had a weak effect on abundance (− 1.25 ± 0.73, z = − 1.72, *p* = 0.08) and richness of overall dung beetles (− 0.72 ± 0.4, z = − 1.79, *p* = 0.07), and the abundance of dweller beetles (− 2.96 ± 1.5, z = − 1.87, *p* = 0.06). The abundance of tunnellers (1.23 ± 0.66, z = 1.85, *p* = 0.06) and Shannon diversity of overall species (0.62 ± 0.35, z = 1.79, *p* = 0.08) were weakly affected by the interaction between habitat and diel period.

### Community composition

**Figure 5:**
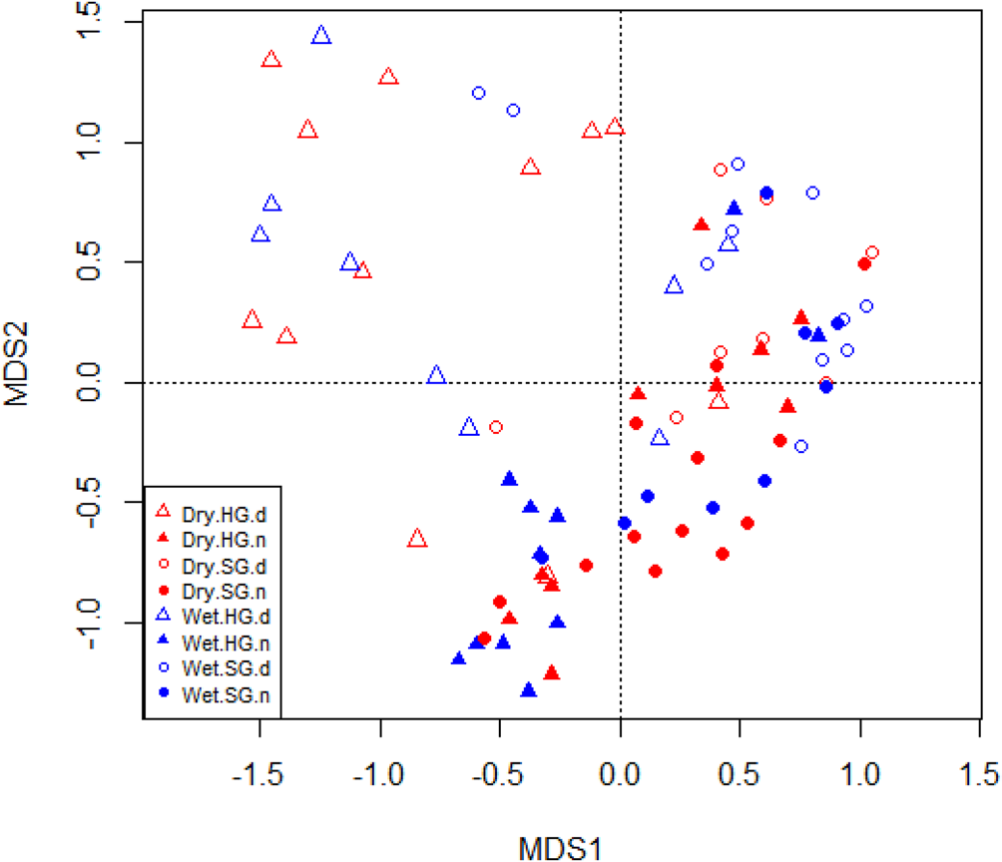
The MDS plot ordinates sites on the season (Dry and Wet), habitat (HG and SG) and diel (*d* day and *n* night) activities of dung beetles. It suggests that the sites, regardless of season and habitat, are relatively closer on nocturnal community of dung beetles than on diurnal community of dung beetles.

The interactions between habitat, season, and diel period (PERMANOVA: *F* = 0.98, *p* = 0.5) and the habitat and season (*F* = 1.76, *p* = 0.08) were not crucial for predicting the community composition of dung beetles. However, habitat (*F* = 4.11, *p* = 0.01), diel period (*F* = 7.81, *p* = 0.01), season (*F* = 3.24, *p* = 0.01), and the interactions of habitat and diel period (*F* = 1.83, *p* = 0.03) and diel period and season (*F* = 2.67, *p* = 0.02) influenced the community composition of dung beetles. The group dispersion was not significant for the season (PERMDISP: *F* = 0.19, *p* = 0.6) and the habitat (*F* = 0.73, *p* = 0.39), but was significant for the diel period (*F* = 9.73, *p* = 0.02) (Fig. 5).

**Figure 6:**
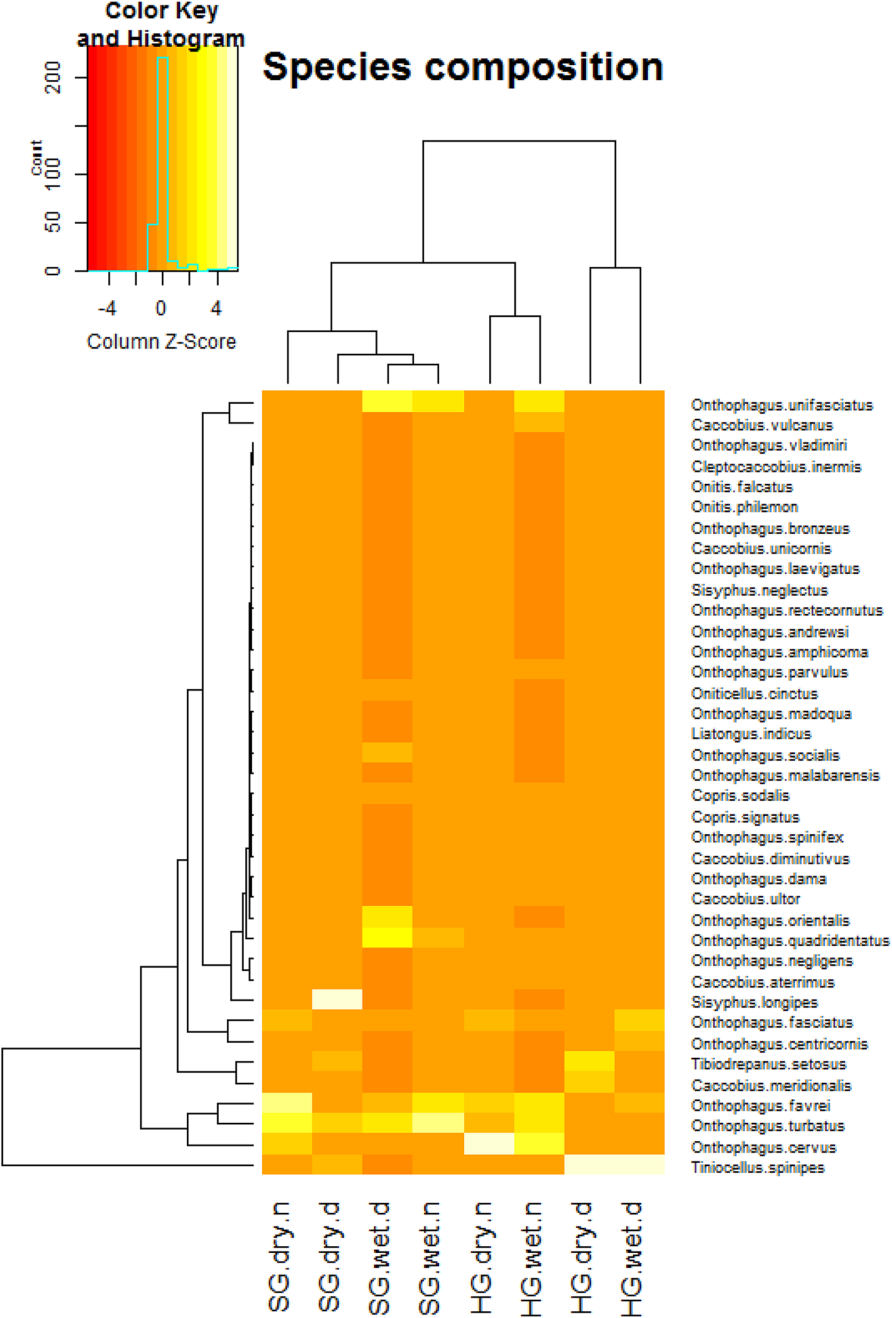
Heat map shows that the diurnal community of open habitat (HG) by any season is distinct from other communities in open (HG) and closed habitats (SG). *dry and wet* dry season and wet season, *d and n* diurnal and nocturnal.

The sites were primarily ordered by the diel activity periods—day and night (Fig. 5). The sites came closer on the nocturnal community of dung beetles in both the seasons (Fig. 5). The sites, however, dispersed relatively more on the diurnal community of dung beetles (Fig. 5). The heatmap brought out two significant groups of sites based on the dung beetle community: first, a diurnal open habitat group and second, a nested closed habitat-nocturnal open habitat group (Fig. 6). The community structure of dung beetles in the habitats suggest that some dung beetles of closed habitats might explore the open habitat during night for foraging dung.

The dominant species of both the habitats were different during diurnal and nocturnal periods of the day and in the habitats (Table 2). In HG, *T. spinipes* and *O. cervus* were the respective dominant species of day and night in dry and wet seasons. In SG, *O. turbatus* and *O. favrei* were the respective dominant species during day and night in the dry season. In the wet season, *O. quadridentatus* replaced *O. turbatus* during the day, and *O. turbatus* replaced *O. favrei* during the night. The diurnal species of open habitat hardly used it during the night and the closed habitat in any seasons. *Tiniocellus spinipes* with 712 individuals (range = 4–440) was the most dominant species of HG during the day, which, however, was represented by just three individuals during the night in HG; in SG, eight and two individuals of this species were collected respectively during day and night . *Tibiodrepanus setosus* had 145 individuals in HG during the day; at night, it sampled just one individual; the SGs sampled five and no individuals during day and night, respectively. *Onthophagus fasciatus* was represented by 139 (day) and 21 individuals (night) in HG, and five (day) and 15 individuals (night) in SG. *Caccobius meridionalis* had a distribution of 129 and zero individuals in HG during day and night respectively; in SG, it was collected by two individuals each during day and night (Fig. 6, Table 2). The heatmap shows that these diel indicator species brought out by the IndVal were pivotal for grouping the sites (Fig. 6).

**Table 2:** Rank abundance of dung beetles in open (HG) and closed (SG) habitats over diel periods. The bolded are some species that showed contrast activities across habitats and by the diel periods. *HGD* home garden diurnal, *HGN *home garden nocturnal, *SGD* sacred grove diurnal, *SGN* sacred grove nocturnal.

Species	HGD	Species	HGN	Species	SGD	Species	SGN
***Tiniocellus spinipes* (Roth, 1851)**	**712**	*Onthophagus cervus* (Fabricius, 1798)	279	*Sisyphus longipes* (Oivier, 1789)	40	*Onthophagus turbatus* (Walker, 1858)	89
***Tibiodrepanus setosus* (Wiedemann, 1823)**	**145**	*Onthophagus favrei* (Boucomont, 1914)	137	*Onthophagus turbatus* (Walker, 1858)	20	*Onthophagus favrei* (Boucomont, 1914)	88
*Onthophagus fasciatus* (Boucomont, 1914)	139	*Onthophagus turbatus* (Walker, 1858)	121	*Onthophagus unifasciatus* (Schaller, 1783)	10	*Onthophagus cervus* (Fabricius, 1798)	29
***Caccobius meridionalis*** (Boucomont, 1914)	**129**	*Onthophagus unifasciatus* (Schaller, 1783)	100	*Onthophagus quadridentatus* (Fabricius, 1798)	8	*Onthophagus unifasciatus* (Schaller, 1783)	21
***Onthophagus centricornis* (**Fabricius, 1798)	**115**	*Caccobius vulcanus* (Fabricius, 1801)	59	***Tiniocellus spinipes* (Roth, 1851)**	**8**	*Onthophagus fasciatus* (Boucomont, 1914)	15
*Onthophagus favrei* (Boucomont, 1914)	99	*Onthophagus negligens* (Walker, 1858)	24	*Onthophagus orientalis* (Harold, 1868)	6	*Onthophagus quadridentatus* (Fabricius, 1798)	10
*Onthophagus turbatus* (Walker, 1858)	47	*Caccobius aterrimus* (Fabricius, 1798)	22	*Onthophagus fasciatus* (Boucomont, 1914)	5	*Onthophagus negligens* (Walker, 1858)	9
*Onthophagus unifasciatus* (Schaller, 1783)	40	*Onthophagus fasciatus* (Boucomont, 1914)	21	***Tibiodrepanus setosus* (Wiedemann, 1823)**	**5**	*Caccobius vulcanus* (Fabricius, 1801)	8
*Caccobius vulcanus* (Fabricius, 1801)	25	*Onthophagus dama* (Fabricius, 1798)	5	*Onthophagus favrei* (Boucomont. 1914)	4	***Caccobius meridionalis*** (Boucomont, 1914)	**2**
*Onthophagus cervus* (Fabricius, 1798)	21	*Copris signatus* (Walker, 1858)	4	***Caccobius meridionalis*** (Boucomont, 1914)	**3**	*Copris signatus* (Walker, 1858)	2
*Oniticellus cinctus* (Fabricus, 1775)	6	*Copris sodalis* (Walker, 1858)	4	*Onthophagus andrewesi* (Arrow, 1931)	3	*Onthophagus centricornis* (Fabricius, 1798)	2
*Onthophagus malabarensis* (Boucomont, 1919)	6	*Onthophagus spinifex* (Fabricius, 1781)	4	*Oniticellus cinctus* (Fabricus, 1775)	2	***Tiniocellus spinipes* (Roth, 1851)**	**2**
*Onthophagus parvulus* (Fabricius, 1798)	5	*Caccobius diminutivus* (Walker, 1858)	3	*Onthophagus amphicoma* (Boucomont, 1914)	2	*Caccobius unicornis***(**Fabricius, 1798)	1
*Onthophagus dama* (Fabricius, 1798)	4	*Caccobius ultor* (Sharp, 1875)	3	***Onthophagus centricornis* (**Fabricius, 1798)	**2**	*Copris sodalis* (Walker, 1858)	1
*Onthophagus madoqua* (Arrow, 1931)	4	***Tiniocellus spinipes* (Roth, 1851)**	**3**	*Onthophagus cervus* (Fabricius, 1798)	2	*Onthophagus amphicoma* (Boucomont, 1914)	1
*Onthophagus socialis* (Arrow, 1931)	4	*Onthophagus parvulus* (Fabricius, 1798)	2	*Onthophagus socialis* (Arrow, 1931)	2	*Onthophagus parvulus* (Fabricius, 1798)	1
*Caccobius ultor* (Sharp, 1875)	3	*Onthophagus quadridentatus* (Fabricius, 1798)	2	*Copris sodalis* (Walker, 1858)	1	*Onthophagus rectecornutus* (Lansberge, 1883)	1
*Liatongus indicus* (Arrow, 1908)	3	*Onitis falcatus* (Wulfen, 1786)	1	*Onthophagus bronzeus* (Arrow, 1907)	1	*Onthophagus socialis* (Arrow, 1931)	1
*Onthophagus andrewesi* (Arrow, 1931)	3	*Onitis philemon* (Fabricius, 1801)	1	*Onthophagus parvulus* (Fabricius, 1798)	1		
*Onthophagus rectecornutus* (Lansberge, 1883)	2	***Onthophagus centricornis* (**Fabricius, 1798)	**1**	*Onthophagus rectecornutus* (Lansberge, 1883)	1		
*Cleptoaccobius inermis* (Arrowi, 1931)	1	*Onthophagus laevigatus *(Fabricius, 1798)	1				
*Onitis falcatus* (Wulfen, 1786)	1	*Onthophagus orientalis *(Harold, 1868)	1				
*Onthophagus amphicoma* (Boucomont, 1914)	1	***Tibiodrepanus setosus* (Wiedemann, 1823)**	**1**				
*Onthophagus orientalis *(Harold, 1868)	1						
*Onthophagus vladimiri* (Frey, 1957)	1						
*Sisyphus longipes* (Oivier, 1789)	1						
*Sisyphus neglectus* (Gory, 1833)	1						

## Discussion

We found a higher activity of dung beetles in relatively open habitats than the closed island forests of the sacred groves in the tropical village landscape of peninsular India. Season, neither as a stand-alone factor nor in interaction with any other factors (diel period and habitat), predicted richness, abundance, or diversity of dung beetles, but predicted the abundance activity of tunnellers and rollers. The tunnellers were sampled more during the wet season than the dry season. The rollers, on the other hand, were sampled only during the dry season. The diel period—day and night period—did not affect the overall abundance or richness of the beetles, but affected the activities of dweller and roller communities. All rollers and most of the dwellers were mainly collected during the day. The effect of the diel period is more prominent on the dominant species of open habitat than on the dominant species of closed habitat. However, the study found that the abundance activity of overall dung beetles in open and closed habitats can be different with the diel period of the day. In the closed habitats, the dung beetles were more active during the night than during the day. In open habitats, the abundance and richness activities of the dung beetles were not affected. This contrasts with our expectations that the temperature is a significant predictor of overall dung beetle activities. However, the species turned over along the gradients of habitats, diel periods, seasons, and by the paired interactions of these factors. The nocturnal community of dung beetles of the open habitat of both the dry and wet seasons was similar to the community of closed habitat, but the diurnal community of dung beetles of the open habitat of both the seasons stood out from the other communities.

Except for the desert species, the coprophagous dung beetles generally have a diurnal and a nocturnal community^48^. Sacred groves in the study system are distributed in a matrix of human settlements and home gardens as small closed semi-evergreen forest islands. This allows the dung beetle species to opt for a particular habitat or a microhabitat—a closed one and an open one, and a diel period—day and night—for their activities. The beetles that are intolerant to temperature can interact with the habitat for their activities. The sampling units in the paired habitats stood within a 300 m radius in all the eleven sites. This allows us understanding how dung beetles respond to two contrasting habitats during diurnal and nocturnal periods. We have sufficiently replicated the HG–SG pair in the study system. Therefore, various trends that we see on the activity of dung beetles in habitats, diel periods, seasons, and their interactions can be extrapolated with a high degree of confidence. However, we caution that the findings might be relevant for an island forest-home garden matrix system of a village landscape, such as sacred groves-home gardens of the present study. Studies in forest landscapes that have closed continuous forest tracts and open grasslands or agrolandscapes can further our understanding on the diel activity of dung beetles in association with the habitats and seasons.

Dung beetles were abundant, rich, and diverse in open habitat (HG) than the closed habitat (SG), a pattern some previous studies have illustrated for other tropical parts of the world^17,49,50^. There are both a set of environmental and dung beetle-specific biological factors postulated as the reasons for such a pattern in habitats on the closed-open gradients^16,17,51^. It is suggested that the volatiles emitting from the dung have a far better reach and sensed well by the beetles in open habitats than the closed habitats^16,52^. The relatively high radiant temperature of the soil in open habitat during the day favors flight activity of dung beetles^16,51^. The habitats we studied were different in day temperature, light intensity or shade, and vegetation. The differences in abundance, richness, Shannon diversity, and community structure of dung beetles in open and closed habitats suggest that the dung beetles might be partitioning their activities on the shade level and the temperature gradients of the habitats. The strikingly different communities of dung beetles in the day and night periods of open habitat also suggest that the beetles segregate into communities over the temperature gradients. The open habitat specialists hardly used the closed habitat for foraging the dung under any circumstance of day or night of dry or wet seasons. They even found the open habitat far less amenable during the night. These findings contrast with the findings that the dung beetle species may have biological mechanisms to cope with the temperature fluctuations along the spatiotemporal contexts^23,53^.

The patterns of dung beetle abundance of day and night periods varied with the habitats. In open habitat, day and night supported a similar abundance of dung beetles. This contrasts with the findings of Iannuzzi et al.^54^ and da Silva et al.^31^, who found a higher abundance activity of dung beetles in open habitats during day in Neotropical climates of Brazil. In closed habitats, da Silva et al.^31^ found a higher abundance of dung beetles during the day, but Iannuzzi et al.^54^ found a higher abundance of dung beetles during the night. In our study, the nocturnal abundance of dung beetles was higher than diurnal abundance in closed habitats, a pattern Iannuzzi et al.^54^ reported for Neotropical lowlands. The different patterns of dung beetle abundance that we see along spatiotemporal gradients could be due to the dominant species participating in the respective communities. Although our findings agree entirely or partly with the Neotropical studies^31,54^, we caution that our study system that includes the natural sources of dung, habitat types, fragment sizes of habitat, and taxonomic composition of dung beetles, and the dung beetle baiting methods, is different from the studies in Neotropics that we referred here.

Our study reveals that the study region has two different beetle communities sharing resources on two diel periods. Open habitat had its own diurnal and nocturnal communities for sharing dung (Fig. 3). It had a diurnal community with the clear dominance of four diurnal species—*T. setosus*, *T. spinipes*, *O. centricornis*, and *C. meridionalis*, and a nocturnal community constituted primarily by two nocturnal species—*C. aterrimus* and *O. cervus*—and many diurnal-nocturnal species (Fig. 6). It is intriguing that the two signature species of day period of open habitat—*T. setosus* and *T. spinipes*—were almost missing in the nocturnal dung pads of both the open and closed habitats. It seems the night temperature or the darkness impairs their nocturnal foraging or movement, a potential topic for a future study. However, no such distinct diel communities with nocturnal or diurnal species have been identified for the closed habitat. It had 24 species of beetles, but 22 were present both in day and night dung pads. Only two common species had their abundance remarkably higher in SG than HG; one was a roller species *S. longipes*—a diurnal species, and another was a tunneller species, *O. quadridentatus*—a species that forage during day and night. Our findings suggest that the dung beetles in the study system, particularly the open habitat species, may not have found the closed system a thermally amenable habitat. The indicator species of the study are open habitat specialists and active during day. However, most of them (*Onthophagus fasciatus*, *Caccobius meridionalis*, *Tibiodrepanus setosus*, *Tiniocellus spinipes*, and *Caccobius vulcanus*) are the characteristic species of the disturbed habitats such as agricultural systems and urban sites^55^.

Dung beetles belong to three functional groups on nesting habits—another adaptation for sharing resources effectively^56–58^. These functional groups are also known to respond to habitat and diel periods. Studies suggest that tunnellers are abundant during the night and rollers are abundant during the day in tropical habitats^31,59^. In the present study, tunneller is species-rich and abundant, a pattern seen in tropical parts of the world^41,60–63^. The study also showed that tunnellers are abundant during the night, dwellers and rollers are abundant during the day, and closed habitat has more rollers than open habitat, and open habitat has more dwellers and tunnellers than closed habitat, a pattern predicted for tropics^31,59,60,63^. Rollers exploit fresh dung as quickly as possible to avoid competition from dwellers and tunnellers^57,64^. Rollers preferred dry season and day period; their larval mortality is reported high in moist and wet dung balls^65–68^, and the day temperature is ideal for rolling activity of the beetles^64,69^.

Moreover, the roller species encountered in the present study is belonging to *Sisyphus*, a genus that requires high temperature and low humidity for nesting^70,71^. The relatively bare floor of HG might have restricted rollers mostly in SG, because the dung balls are stored under the humus layer of topsoil. Conversely, dwellers are abundant in HG. It is suggested that dwellers dominate dung pads of disturbed habitats^7,17,72^. Home gardens in the present study are a disturbed habitat with no leaf litter bed on the ground. The studies suggest that dung beetles of open habitat and diurnal periods are smaller due to thermoregulatory reasons^25,54,59^. Our results partially comply with this finding. Neither HG nor SG sampled larger dung beetles; the largest collected in the present study were *Copris signatus* (15 mm long), *C. sodalis* (15–19 mm long)*, Onitis falcatus* (16–23 mm long)*,* and *O. philemon* (14–19 mm long). Having our sites located on a non-forested village landscape, we are not surprised by the lack of many larger dung beetles. It is suggested that the larger dung beetles are prevalent in forested landscapes that support larger wild mammals, such as elephants and gaurs^42,55,63,73^. However, the agricultural lands in forested landscape can also have the small dung beetle species dominating the communities. In a dung beetle community of Coffee landscape of the Western Ghats, Jayaprakash^73^ collected 30 species of small dung beetles and eight species of big dung beetles.

To conclude, according to the composition of dung beetles in diel periods and seasons, the habitats have been grouped into two: (1) a typical HG day community of both wet and dry seasons and (2) an HG night community of dry and wet seasons nested in the SG community. This suggests that the diurnal community of open habitat is unlikely to be affected by seasonal contrast. It further suggests that the species community of closed habitat may find the open habitat suitable for foraging during the night. Therefore, the diel period—the diurnal-nocturnal period—might affect species community of open habitat, rather than the community of closed habitat. In summary, we found that the dung beetle composition of diurnal and nocturnal communities is different, more so than the differences observed between habitats and seasons. Given that the species composition has turned over between habitats on diel periods and the closed habitat supported remarkably fewer beetles, the closed habitat is unlikely to be a thermal refuge for open habitat species. While we have robust data to support our findings on diel activity and habitat preference of dung beetles, it may be noted that the study was based on a system that has fragmented forests (sacred groves) and orchards in a land-use matrix of a village landscape. Future studies in contiguous forests of different types and grasslands/ agricultural systems can shed more light on diel activity of dung beetles in the Indian tropics.

## Supplementary Information

Supplementary Tables.
