# Supplementary material for: Spatiotemporal effects on dung beetle activities in island forests-home garden matrix in a tropical village landscape
Source: Sci Rep. 2021 Aug 30;11:17398. doi: 10.1038/s41598-021-96831-5 (PMC8405664; doi:10.1038/s41598-021-96831-5)
Supplement: Supplementary file 1 — Supplementary Tables. [file 41598_2021_96831_MOESM1_ESM.docx]

**Electronic Supplementary Materials**

**Spatiotemporal effects on dung beetle activities in island forests-home garden matrix in a tropical village landscape**

G. Asha^$^, K. Manoj, P.P. Megha, Palatty Allesh Sinu*^$^

Table S1. Area of sacred groves in different study sites

| **District** | **Site** | **SG Area** |
| --- | --- | --- |
|  |  |  |
|  |  |  |
| Kasaragod | Cheemeni 1 | 0.026 km^2^ |
|  |  |  |
| Kasaragod | Koyithatta | 0.011 km^2^ |
|  |  |  |
| Kasaragod | Puthiyaparambath | 0.018 km^2^ |
|  |  |  |
| Kasaragod | Edayilakkad | 0.059 km^2^ |
|  |  |  |
| Kasaragod | Kammadam1 | 0.28 km^2^ |
|  |  |  |
| Kasaragod | Kammadam2 | 0.01 km^2^ |
|  |  |  |
| Kasaragod | Mannampurathkavu | 0.021 km^2^ |
|  |  |  |
| Kasaragod | Muthappanarkavu | 0.011 km^2^ |
|  |  |  |
| Kasaragod | Cheermakavu | 0.012 km^2^ |
|  |  |  |
| Kasaragod | Kammadam3 | 0.018 km^2^ |
|  |  |  |
| Kasaragod | Cheemeni 2 | 0.016 km^2^ |
|  |  |  |

Table S2. Species wise abundance in diel periods, habitats, and seasons

|  |  | Diel period | | Habitat | | Seasons | |
| --- | --- | --- | --- | --- | --- | --- | --- |
| Species | Guild | Day | Night | HG | SG | Dry | Wet |
| *Caccobius aterrimus* | Tunneller | 0 | 22 | 22 | 0 | 6 | 16 |
| *Caccobius diminutivus* | Tunneller | 0 | 3 | 3 | 0 | 1 | 2 |
| *Cleptocaccobius inermis* | Tunneller | 1 | 0 | 1 | 0 | 0 | 1 |
| *Caccobius meridionalis* | Tunneller | 132 | 2 | 129 | 5 | 94 | 40 |
| *Caccobius ultor* | Tunneller | 3 | 3 | 6 | 0 | 0 | 6 |
| *Caccobius unicornis* | Tunneller | 0 | 1 | 0 | 1 | 1 | 0 |
| *Caccobius vulcanus* | Tunneller | 25 | 67 | 84 | 8 | 23 | 69 |
| *Copris signatus* | Tunneller | 0 | 6 | 4 | 2 | 0 | 6 |
| *Copris sodalis* | Tunneller | 1 | 5 | 4 | 2 | 0 | 6 |
| *Liatongus indicus* | Dweller | 3 | 0 | 3 | 0 | 0 | 3 |
| *Oniticellus cinctus* | Dweller | 8 | 0 | 6 | 2 | 6 | 2 |
| *Onitis falcatus* | Tunneller | 1 | 1 | 2 | 0 | 0 | 2 |
| *Onitis philemon* | Tunneller | 0 | 1 | 1 | 0 | 0 | 1 |
| *Onthophagus amphicoma* | Tunneller | 3 | 1 | 1 | 3 | 4 | 0 |
| *Onthophagus andrewesi* | Tunneller | 6 | 0 | 3 | 3 | 5 | 1 |
| *Onthophagus bronzeus* | Tunneller | 1 | 0 | 0 | 1 | 1 | 0 |
| *Onthophagus centricornis* | Tunneller | 117 | 3 | 116 | 4 | 35 | 85 |
| *Onthophagus cervus* | Tunneller | 23 | 308 | 300 | 31 | 160 | 171 |
| *Onthophagus dama* | Tunneller | 4 | 5 | 9 | 0 | 0 | 9 |
| *Onthophagus fasciatus* | Tunneller | 144 | 36 | 160 | 20 | 48 | 132 |
| *Onthophagus favrei* | Tunneller | 103 | 225 | 236 | 92 | 116 | 212 |
| *Onthophagus laevigatus* | Tunneller | 0 | 1 | 1 | 0 | 1 | 0 |
| *Onthophagus madoqua* | Tunneller | 4 | 0 | 4 | 0 | 0 | 4 |
| *Onthophagus malabarensis* | Tunneller | 6 | 0 | 6 | 0 | 0 | 6 |
| *Onthophagus negligens* | Tunneller | 0 | 33 | 24 | 9 | 16 | 17 |
| *Onthophagus orientalis* | Tunneller | 7 | 1 | 2 | 6 | 0 | 8 |
| *Onthophagus parvulus* | Tunneller | 6 | 3 | 7 | 2 | 5 | 4 |
| *Onthophagus quadridentatus* | Tunneller | 8 | 12 | 2 | 18 | 1 | 19 |
| *Onthophagus rectecornutus* | Tunneller | 3 | 1 | 2 | 2 | 2 | 2 |
| *Onthophagus socialis* | Tunneller | 6 | 1 | 4 | 3 | 0 | 7 |
| *Onthophagus spinifex* | Tunneller | 0 | 4 | 4 | 0 | 1 | 3 |
| *Onthophagus turbatus* | Tunneller | 67 | 210 | 168 | 109 | 114 | 163 |
| *Onthophagus unifasciatus* | Tunneller | 50 | 121 | 140 | 31 | 20 | 151 |
| *Onthophagus vladimiri* | Tunneller | 1 | 0 | 1 | 0 | 0 | 1 |
| *Sisyphus longipes* | Roller | 41 | 0 | 1 | 40 | 41 | 0 |
| *Sisyphus neglectus* | Roller | 1 | 0 | 1 | 0 | 1 | 0 |
| *Tibiodrepanus setosus* | Dweller | 150 | 1 | 146 | 5 | 128 | 23 |
| *Tiniocellus spinipes* | Dweller | 720 | 5 | 715 | 10 | 334 | 391 |

Table S3. Indicator species of habitats (IndVal index)

| Species | Habitat | stat | p value | length |
| --- | --- | --- | --- | --- |
| *Caccobius vulcanus* | HG | 0.911 | 0.001 | 4.92 |
| *Onthophagus fasciatus* | HG | 0.899 | 0.003 | 5.41 |
| *Tibiodrepanus setosus* | HG | 0.889 | 0.001 | 4.3 |
| *Onthophagus centricornis* | HG | 0.889 | 0.003 | 2.87 |
| *Caccobius meridionalis* | HG | 0.887 | 0.002 | 4.36 |
| *Onthophagus cervus* | HG | 0.861 | 0.053 | 6.43 |
| *Tiniocellus spinipes* | HG | 0.847 | 0.032 | 6.51 |
| *Onthophagus parvulus* | HG | 0.532 | 0.343 | 3 |
| *Caccobius diminutivus* | HG | 0.522 | 0.21 | 3.52 |
| *Oniticellus cinctus* | HG | 0.522 | 1 | 10.46 |
| *Caccobius aterrimus* | HG | 0.426 | 0.462 | 4.03 |
| *Caccobius ultor* | HG | 0.426 | 0.502 | 5.15 |
| *Onitis falcatus* | HG | 0.426 | 0.498 | 18.8 |
| *Onthophagus dama* | HG | 0.426 | 0.484 | 9.83 |
| *Onthophagus madoqua* | HG | 0.426 | 0.482 | 4.29 |
| *Onthophagus malabarensis* | HG | 0.426 | 0.488 | 3.99 |
| *Onthophagus spinifex* | HG | 0.426 | 0.479 | 8.04 |
| *Cleptocaccobius inermis* | HG | 0.302 | 1 | 3 |
| *Liatongus indicus* | HG | 0.302 | 1 | 8.6 |
| *Onitis philemon* | HG | 0.302 | 1 | 18.08 |
| *Onthophagus laevigatus* | HG | 0.302 | 1 | 7.74 |
| *Onthophagus vladimiri* | HG | 0.302 | 1 | 6.6 |
| *Sisyphus neglectus* | HG | 0.302 | 1 | 5.61 |
| *Sisyphus longipes* | SG | 0.516 | 0.221 | 5.88 |
| *Onthophagus quadridentatus* | SG | 0.495 | 0.314 | 6.23 |
| *Onthophagus orientalis* | SG | 0.452 | 0.472 | 7.47 |
| *Caccobius unicornis* | SG | 0.302 | 1 | 2.28 |
| *Onthophagus bronzeus* | SG | 0.302 | 1 | 3.48 |
